# THE LONGITUDINAL PROCESSES LINKING PSYCHOSOCIAL FACTORS AND STRESS-RELATED EXPERIENCES TO HEALTH

**DOI:** 10.1093/geroni/igae098.0552

**Published:** 2024-12-31

**Authors:** Jing Luo, Gabrielle Pfund, Daniel Mroczek

**Affiliations:** Chair; Co-Chair; Discussant

## Abstract

Psychosocial factors, including individual factors (e.g., personality, sense of purpose), contextual factors (e.g., employment status), and stress-related experiences, have been demonstrated as important protective/risk factors of health. However, little is known about the processes and mechanisms linking these factors to health and aging. The current symposium tackles the longitudinal associations between psychosocial factors and health with five presentations investigating the interindividual and intraindividual processes linking these factors to various health outcomes (physical, cognitive, physiological health and well-being) as people age. In the first study, from a life-course perspective, Brown and colleagues examined the effects of low SES and perceived stress in childhood on two physiological health indicators in midlife and the underlying mediators. Zooming into late adulthood, the second study by Choi and Mejía examined the joint effects of spousal combinations of employment status and socioeconomic resources on older couples’ individual and shared manifestations of cumulative health risk. The third study by Chopik examined the prospective effects of subjective well-being on stress reactivity decline and their dynamic reciprocal relations over time at the intraindividual level. The fourth study by Luo and colleagues examined the how the dynamic interplays between personality and people’s stress experiences and health behavior engagement contribute to individual differences in health change. In the fifth study, Pfund and colleagues investigated the dynamic intraindividual associations among sense of purpose, social stress-related perception (loneliness), and cognitive decline over time. Collectively, this set of studies improves our understanding of how psychosocial factors shape people’s health and healthy aging.

